# The Influence of Hyponatremia and Hypokalemia on the Risk of Fractures in Various Anatomical Regions among Adult Trauma Patients: A Propensity Score-Matched Analysis

**DOI:** 10.3390/diagnostics14040355

**Published:** 2024-02-06

**Authors:** Shiun-Yuan Hsu, Cheng-Shyuan Rau, Ching-Hua Tsai, Sheng-En Chou, Wei-Ti Su, Ching-Hua Hsieh

**Affiliations:** 1 Department of Trauma Surgery, Kaohsiung Chang Gung Memorial Hospital, Chang Gung University College of Medicine, Kaohsiung 83301, Taiwan; ah.lucy@hotmail.com (S.-Y.H.); tsai1737@cloud.cgmh.org.tw (C.-H.T.); athenechou@gmail.com (S.-E.C.); s101132@adm.cgmh.org.tw (W.-T.S.); 2 Department of Neurosurgery, Kaohsiung Chang Gung Memorial Hospital, Chang Gung University College of Medicine, Kaohsiung 83301, Taiwan; ersh2127@adm.cgmh.org.tw

**Keywords:** trauma, hyponatremia, hypokalemia, fall, fracture

## Abstract

Background: Hyponatremia and hypokalemia are common electrolyte imbalances in trauma patients and have been identified to be risk factors for a fall. In addition, hyponatremia was reported to be related to osteoporosis and fragility fractures, while the association between hypokalemia and osteoporosis has only been reported in rare case reports. This study investigated the impact of hyponatremia and hypokalemia on the incidence of fractures in various body regions of adult trauma patients, using the propensity score-matched patient cohort to reduce the influence of patients’ baseline characteristics. Methods: The study analyzed data from 11,173 hospitalized adult trauma patients treated from 1 January 1998, to 31 December 2022. The study included 1968 patients with hyponatremia and 9205 without, and 1986 with hypokalemia and 9187 without. Different 1:1 propensity score-matched cohorts were generated to create the 1903 pairings of patients with or without hyponatremia, 1977 pairings of patients with or without hypokalemia, and 380 pairing of patients with both hyponatremia and hypokalemia vs. normal control patients. Analysis was conducted on the incidence of fracture in various anatomic regions. Results: Hyponatremic patients had increased odds of thoracic vertebral fracture [odds ratio (95% confidence interval) 1.63 (1.10–2.42), *p* = 0.014], pelvic fracture [2.29 (1.12–4.67), *p* = 0.019], and femoral fracture [1.28 (1.13–1.45), *p* < 0.001] but decreased odds of radial and patella fractures. Hypokalemic patients showed no significant differences in fracture risk except for a decreased likelihood of radial fractures. The patients with both hyponatremia and hypokalemia showed a decreased likelihood of radial fractures and patella fractures. Conclusion: Hyponatremia may have a greater impact on the occurrence of bone fractures than hypokalemia in trauma patients who have suffered a fall. Electrolyte abnormalities should be taken into account while assessing the risk of fractures in trauma patients.

## 1. Introduction

Chronic hyponatremia has been linked to an increase in the incidence of falls and fractures [1]. Studies have shown that even mild hyponatremia can lead to gait disturbances, decreased mentation, and falls, especially from walking height or less than one meter [2,3]. Hyponatremia appears to contribute to falls and fractures through two mechanisms [4]. Initially, it induces a state of moderate cognitive impairment, leading to instability in walking and an increased likelihood of experiencing falls. This can likely be attributed to the depletion of glutamate, a neurotransmitter that plays a crucial role in the regulation of gait function, as a consequence of the brain’s adaptation to prolonged hyponatremia [4]. This association is further supported by findings from De Giorgi et al. [5] and Tolouian et al. [6], who noted that mild hyponatremia is linked to unsteadiness and attention deficits, which are risk factors for falls. Even modest chronic hyponatremia has been shown to impair cognitive performance and increase the risk of falling [7,8]. Second, hyponatremia increases the risk of osteoporosis and bone fragility by increasing bone resorption to mobilize salt reserves in bone.

A significant relationship between hyponatremia and osteoporosis has been established. Kruse, Eiken, and Vestergaard [9] discovered that hyponatremia is linked to reduced bone mineralization in the hip as well as an increased risk of osteoporosis. A significant odds ratio for fractures, in addition to a substantial correlation between hyponatremia, osteoporosis, and fractures, has been observed in previous research [10,11]. Multivariate conditional logistic regression models demonstrated that hyponatremia was associated with osteoporosis and fragility fractures [12]. Additionally, hyponatremia is linked to disordered osteoclast and osteoblast activity, affecting bone health [12]. Low levels of circulating sodium directly boost osteoclastogenesis and bone resorption activity by lowering the uptake of ascorbic acid by cells and increasing oxidative stress. These effects depend on the amount of sodium present [4]. Therefore, chronic hyponatremia leads to increased osteoclast numbers and resorptive activity, resulting in resorptive osteoporosis [13]. 

Hypokalemia, a condition characterized by low potassium levels in the blood, has been identified as a risk factor for falls, particularly in the elderly and in situations involving falls from walking height or less than one meter. The condition can lead to muscle weakness, fatigue, and dizziness, which are all factors that can increase the likelihood of falls. Additionally, hypokalemia has been associated with various cardiovascular complications, such as arrhythmias [14] or electrolyte disturbances [15], can further contribute to the risk of falls. Contrastingly, reports for hyponatremia, the relationship between hyperkalemia and osteoporosis is not established in the literature and is only seen in a few case reports. A case report has shown that hypokalemia is secondary to hypomagnesium, which is also associated with hypoparathyroidism with the suppression of bone remodeling [16]. The hypokalemia associated with a significant prevalence of osteoporosis had been reported in the patients with chronic pancreatitis [17]. In these chronic pancreatitis patients, the chronic relapsing nature of the disease process in the pancreas result in maldigestion with malabsorption of vitamin D and calcium and thus lead to hypokalemia [17]. Gitelman syndrome is an autosomal recessive hereditary illness that is characterized by several clinical manifestations, including hypokalemia, metabolic alkalosis, hypomagnesemia, and hypocalciuria. Notably, it has been observed that individuals with Gitelman syndrome also have high bone mineral density, thus highlighting the association between hypokalemia and this particular skeletal characteristic [18]. In individuals presenting with incomplete Sjogren’s syndrome, the presence of hypokalemia has been seen to be correlated with persistent metabolic acidosis. This condition leads to enhanced release of alkaline substances from the skeletal system, hence fostering the progression of osteoporosis [19].

The correlation between hyponatremia or hypokalemia and an elevated risk of fractures might be confounded by some factors, such as age [20]. The increased risk of falls among patients with hyponatremia or hypokalemia is especially concerning in the elderly, who are already at an increased risk of falls due to other age-related factors, according to Tachi et al. [21]. Additionally, certain authors have suggested that hyponatremia and hypokalemia should be regarded as indicators of compromised health rather than as isolated risk factors for fractures [20]. Furthermore, although hip fractures are consistently identified as the most prevalent among patients with hyponatremia [4,22,23,24,25], no distinct order is established regarding the ranking of other fracture sites. Furthermore, there are also no published reports concerning the location of fractures associated with hypokalemia. As a result, this study aimed to assess the frequency of fractures in various body regions in hospitalized adult trauma patients with hyponatremia or hypokalemia using a propensity score-matched patient cohort to decrease inequalities in baseline factors such as age, sex, and comorbidities.

## 2. Materials and Methods

### 2.1. Declaration of Ethics

The study employed a retrospective cross-sectional analytic approach, utilizing data from the trauma registry system at Chang Gung Memorial Hospital. This research has been granted permission by the Institutional Review Board (IRB) of the hospital, with the authorization number 202100761BAS. According to the regulation of IRB, the research was given an exemption from the informed consent procedure.

### 2.2. Criteria for Selection of Patients wih Hyponatremia or Hypokalemia

**Figure 1:**
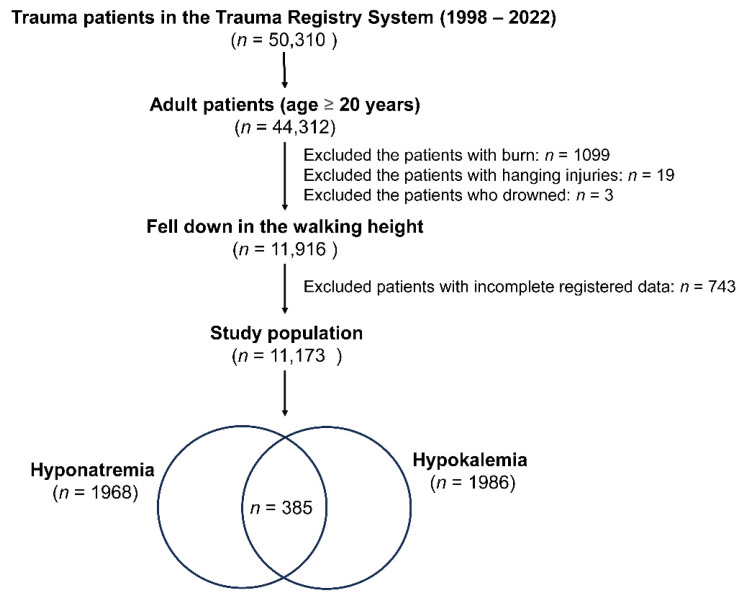
A flowchart depicting the process of selection of hospitalized adult trauma patients with hyponatremia and/or hypokalemia from the trauma registered database.

In this research, as shown in Figure 1, of a total of 50,310 trauma patients in the Trauma Registry System (1998–2022), only adult patients aged 20 years or older were included, amounting to 44,312 individuals. Only those people who fell down while walking were considered for this study (a total of 11,916 patients). Exclusions were made based on specific injury types and health conditions: 1099 patients with burn injuries, 19 with hanging injuries, and 3 who drowned. Additionally, 743 patients with incomplete registered data were excluded. The blood collected for detecting the electrolyte level of these fall patients was performed at the time of arrival to the emergency room. Hyponatremia is characterized by a serum sodium concentration below the threshold of 135 mEq/L [26], whereas hypokalemia is defined as a potassium level lower than or equal to 3.5 mEq/L [27]. After applying these criteria, the final study population comprised 11,173 patients, including 1968 cases of hyponatremia, 1986 cases of hypokalemia, and 385 cases with both hyponatremia and hypokalemia.

### 2.3. Gathering of Clinical Information

The trauma registry system was utilized to gather a multitude of medical details. The information included in this document comprised patient demographics, including age and sex, as well as a record of any pre-existing medical conditions the patient had, such as diabetes mellitus (DM), hypertension (HTN), cerebrovascular accident (CVA), coronary artery disease (CAD), congestive heart failure (CHF), and end-stage renal disease (ESRD). Injury severity was evaluated using the Glasgow Coma Scale (GCS) and the Injury Severity Scores (ISS). The location of patients’ fractures was documented. The length of each patient’s hospitalization, expressed in days, was documented, along with any instances of mortality that occurred within the facility.

### 2.4. Statistical Analyses

The analytical assessments were performed using the SPSS 23.0 program, developed by IBM Corp. (Armonk, NY, USA) and tailored for the Windows operating system. The Kolmogorov–Smirnov test was used to evaluate the normalization of scattered data relevant to continuous variables. The correlation between continuous variables was initially examined using a one-way analysis of variance (ANOVA). Subsequently, Games–Howell post-hoc tests were employed to further investigate any significant differences identified in the ANOVA. The test findings are reported in the form of a mean value accompanied by its matching standard deviation. The examination of categorical variables entailed the utilization of either Fisher’s exact tests or Pearson’s chi-squared (χ^2^) tests. The researchers computed odds ratios (ORs) and their accompanying 95% confidence intervals (CIs).

In order to effectively account for any initial differences in baseline characteristics among different patient groups, especially when evaluating the incidence of fractures in different anatomical regions, a cohort with a 1:1 propensity score matching was created using the NCSS software version 10 (NCSS LLC, Kaysville, UT, USA), which was developed by the NCSS Statistical program located in Kaysville, Utah. The approach utilized in this investigation was the implementation of the Greedy strategy, with a caliper width of 0.2. The propensity scores that were utilized in this study were obtained by the implementation of a logistic regression model, which considered several parameters such as gender, age, and pre-existing medical conditions. To identify statistically significant differences between groups, a predetermined threshold for the *p*-value was established at a level below 0.05.

## 3. Results

### 3.1. Characteristics of the Patients with Hyponatremia

For hyponatremia, the study involved 1968 and 9205 patients with and without hyponatremia. As shown in the Table 1, the statistics demonstrate that these groups differ significantly in terms of sex, age, and various comorbidities. Males made up a lower proportion of the hyponatremia group (40.5%) compared with 35.4% of the patients without hyponatremia. The average age in the hyponatremia group was substantially greater (74.2 years) than in the non-hyponatremia group (68.0 years). Comorbidities like cerebrovascular accident (CVA), hypertension (HTN), coronary artery disease (CAD), congestive heart failure (CHF), diabetes mellitus (DM), and end-stage renal disease (ESRD) were more prevalent in the hyponatremia group, with ORs ranging from 1.47 for CVA to 2.58 for ESRD. Glasgow Coma Scale (GCS) scores and Injury Severity Score (ISS) also differed significantly, indicating a more severe injured status in the hyponatremia group. Mortality rates were higher in patients with hyponatremia than those without (4.9% vs. 2.0%, respectively, *p* < 0.001). The hospital stay was significantly longer in those patients with hyponatremia than those without (10.1 days vs. 7.1 days, respectively, *p* < 0.001).

### 3.2. The Fracture Risks of Location in Patients with Hyponatremia

For patients with and without hyponatremia, a propensity score-matched patient cohort of 1:1 was established to reduce the influence of confounding factors related to the patients’ baseline characteristics on outcome assessments. The propensity score-matched patient populations, comprising 1903 pairings, exhibited no statistically significant variations in terms of age, comorbidities, or sex (Table 2). Hyponatremia greatly increased the risk of sustaining a thoracic vertebral fracture [OR (95% CI) 1.63 (1.10–2.42), *p* = 0.014], pelvic fracture [2.29 (1.12–4.67), *p* = 0.019], and femoral fracture [1.28 (1.13–1.45), *p* < 0.001] compared with those without hyponatremia (Table 3). However, they had lower odds of sustaining a radial fracture [0.63 (0.51–0.79), *p* < 0.001] and a patella fracture [0.43–0.92), *p* = 0.017] than patients without hyponatremia.

### 3.3. Characteristics of the Patients with Hypokalemia

In the case of hypokalemia, the study involved 1986 and 9187 patients with and without hypokalemia (Table 4). The results show no significant differences in sex and age. Comorbidities like CVA and HTN were more prevalent in the hypokalemia group, with ORs ranging from 1.22 for CVA to 1.30 for HTN. However, the DM and ESRD were less prevalent in the patients with hypokalemia than those without, with ORs ranging from 0.73 for DM to 0.70 for ESRD. A significantly lower GCS score but higher ISS indicated a higher injury severity in the hypokalemia group. In patients with hypokalemia, the mortality rate was significantly higher at 4.2% compared with 2.2% in the absence of hypokalemia (*p* < 0.001). Patients with hypokalemia had a substantially longer hospital stay (8.7 days vs. 7.4 days, respectively, *p* < 0.001) than those without hypokalemia.

### 3.4. The Fracture Risks of Location in Patients with Hypokalemia

A cohort of 1977 patient pairings, including those with and without hypokalemia, was chosen in accordance with a propensity score match ratio of 1:1. There were no significant statistical differences identified with regard to age, sex, or comorbidities (Table 5). The likelihood of developing a radial fracture was found to be significantly reduced in patients with hypokalemia [0.66 (0.55–0.80), *p* < 0.001] compared with those without hypokalemia (Table 6). Regarding the incidence of fractures in other body regions, no significant statistical distinction was observed between patients who had hypokalemia and those who did not.

### 3.5. Characteristics of the Patients with Both Hyponatremia and Hypokalemia

This study comprised 385 patients who presented with both hyponatremia and hypokalemia. In contrast, 7601 patients were included as the normal control group for subsequent analysis, devoid of either hyponatremia or hypokalemia (Table 7). In addition to age and sex, the results indicate that these groups differ substantially with regard to a variety of comorbidities, such as HTN, CAD, DM, and ESRD, all of which were more prevalent among patients who presented with hyponatremia and hypokalemia. In patients with both hyponatremia and hypokalemia, a substantially lower GCS score but a higher ISS score indicated a more severe injury. Mortality was significantly higher at 7.3% in patients with both hyponatremia and hypokalemia compared with 1.7% in normal patients (*p* < 0.001), and hospital stays were substantially longer (11.6 days vs. 6.9 days, respectively, *p* < 0.001).

### 3.6. The Fracture Risks of Location in Patients with Both Hyponatremia and Hypokalemia

A cohort of 380 patient pairings, including those with both hyponatremia and hypokalemia vs. those normal patients, was chosen in a propensity score match ratio of 1:1. There were no significant statistical differences identified with regard to age, sex, or comorbidities. (Table 8). The likelihood of developing a radial fracture was found to be significantly reduced in patients with both hyponatremia and hypokalemia [0.50 (0.31–0.80), *p* = 0.004] compared with those normal patients (Table 9). Regarding the incidence of fractures in other body regions, no significant statistical distinction was observed.

## 4. Discussion

Following propensity score matching to account for differences in baseline characteristics, this research demonstrated that individuals with hyponatremia have an increased risk of sustaining a thoracic vertebral fracture, pelvic fracture, and femoral fracture in comparison with those without hyponatremia. However, hypokalemia did not show a similar association. In addition, hyponatremia is correlated with a decreased likelihood of developing a radial fracture and a patella fracture, whereas hypokalemia is associated with decreased likelihood of developing a radial fracture. A summary of the risk of bone fractures in adult trauma patients who are hospitalized and have hyponatremia and/or hypokalemia was provided in Table 10.

This study’s results indicated that hyponatremia was linked to thoracic vertebral, pelvic, and femoral fractures when someone falls. The study results are in accordance with those reports describing the patients with hyponatremia who have experienced a fall had the pelvis and femur as the most common sites of fracture [2,6]. In addition to an elevated risk of hip fracture, hyponatremia is also linked to a higher incidence of morphometric spine fractures and incident morphometric spine fractures [28,29]. Chronic hyponatremia increases the prevalence of vertebral fractures after low-energy trauma due to decreased bone quality [30]. Additional research suggests that individuals with central fractures demonstrate reduced bone mineral density, a poorer trabecular bone score, and a greater incidence of vertebral fractures in comparison with individuals with peripheral fractures [31]. A complex and varied relationship between fractures in different parts of the skeleton after a fall might be influenced by factors not only like osteoporosis or bone mineral density, but also the specific bones involved. A study using cadaveric specimens to directly measure experimental bone strength at L4 vertebra and proximal femur sites as the reference standard and to determine the correlations with strength at the radius and tibia [32], indicating that the increased fractures in central and large bones are related to osteoporosis, while fractures in peripheral bones are not [33]. And this is the reason why a peripheral bone mineral densitometry strategy using forearm bone mineral density alone will miss many individuals with osteoporosis [34]. Although the electrolytes imbalance might cast influence on the bone mineral density; however, in this study, the incidences of radial fracture were decreased in the patients with either hyponatremia or hypokalemia. Notably, although these patients might be more prone to certain types of injuries (like vertebral fractures) due to their overall health status, while being less exposed to the mechanisms that commonly cause bony fractures. Patients with hyponatremia often have accompanying symptoms such as muscle weakness, fatigue, or neurological changes. These symptoms can lead to a decrease in physical activity levels, leading to a different type of fracture during the fall that typically result in radial fractures.

Gender differences significantly influence fracture patterns and locations resulting from falls. Females, especially those who have reached the postmenopausal stage, have a greater susceptibility to hip fractures as a result of a heightened incidence of osteoporosis and diminished bone mineral density in comparison with males [35]. This is further exacerbated by age-related changes in body composition and hormonal changes [36,37]. Men, on the other hand, are more likely to experience upper limb fractures, such as those of the humerus and clavicle, attributed to differences in fall mechanics and protective responses during falls [38]. These gender-specific fracture patterns underscore the importance of targeted prevention strategies, considering the distinct anatomical and physiological differences between men and women. It has been found that the incidence of vertebral fracture increases with age in both men and women, however, the increase is greater in women than in men [39]. Differential bone microstructure is linked to vertebral fractures in both men and women, but the particular causes differ: trabecular bone density at the tibia in women and femoral neck areal bone mineral density in males are linked to vertebral fractures separately [40]. Although peripheral bone strength can predict axial bone quality, the association is not as strong in women as it is in males [41].

In addition, comorbidities can significantly influence fracture patterns and locations in falls. For example, individuals with DM often have a higher risk of lower limb fractures, particularly of the foot and ankle, due to diabetic neuropathy and vascular complications affecting bone quality [42]. Patients with HTN and CAD may experience falls due to dizziness or weakness, leading to a higher incidence of hip and upper extremity fractures [43]. ESRD patients, often with altered bone metabolism, are more susceptible to hip and vertebral fractures [44]. CHF patients, due to their reduced physical activity and associated osteoporosis, are at an increased risk of vertebral and hip fractures [45]. Lastly, individuals with a history of CVA have a predisposition to falls and fractures, particularly on the affected side, due to hemiparesis and balance issues [46].

By utilizing a propensity score-matched patient cohort, this research aims to analyze fracture incidents while mitigating the influence of confounding factors including age, sex, and comorbidities. This study reported that hyponatremia is associated with increased incidences of fractures of thoracic vertebral fracture, pelvic fracture, and femoral fracture, which may be caused by hyponatremia’s influence on reducing bone mineralization, which has been linked with an elevated risk of osteoporosis. While such adverse effects were not observed in hypokalemic individuals, further research is required to examine the long-term effects of hyponatremia and hypokalemia, as well as to investigate the underlying mechanisms that contribute to these conditions affecting bone health and fracture risk. It is also recommended that this research be expanded to encompass diverse patient populations and geographic regions to ascertain whether the observed associations between hyponatremia, hypokalemia, and fracture risk are consistent in different demographic and environmental contexts.

This study has substantial limitations. First, the retrospective approach, in combination with the removal of missing data, may cause selection bias. Second, the usage of drugs and nutritional state in this study population are unclear, which may contribute to bias in the interpretation of the data. Third, some diseases, such as hypophosphatemia or metabolic alkalosis [47], syndrome of inappropriate antidiuretic hormone secretion (SIADH) [11,25], primary hyperaldosteronism [48,49], Cushing syndrome [50,51,52], and Bartter syndrome [53], may be strongly linked to hyponatremia or hypokalemia and fracture incidence but were not excluded or analyzed in this study. Finally, the study is confined to a single trauma center, potentially limiting the generalizability of its findings to broader populations. Therefore, caution should be exercised when attempting to apply our results to different geographical contexts. 

## 5. Conclusions

This study revealed a correlation between hyponatremia and a higher occurrence of fractures in the thoracic vertebra, pelvic, and femoral bones, while a lower occurrence of fractures was observed in the radial and patella bones. However, patients with hypokalemia were found to be merely related with a decreased risk of radial fracture. The hyponatremia may exert a more significant influence on the occurrence of bone fractures compared with the hypokalemia observed in trauma patients who have had a fall. Electrolyte abnormalities should be taken into account while evaluating the risk of fractures in trauma patients. Furthermore, it is advisable to regularly monitor the levels of electrolytes, namely sodium, in those patients who are at risk of falling.

## Figures and Tables

**Table 1:** Demographic and injury characteristics of adult trauma patients admitted to the hospital—a comparison with and without hyponatremia.

	Hyponatremia			
Variables	Yes *n* = 1968	No *n* = 9205	OR (95% CI)	*p*
Sex				<0.001
Male, *n* (%)	798 (40.5)	3257 (35.4)	1.25 (1.13–1.38)	
Female, *n* (%)	1170 (59.5)	5948 (64.6)	0.80 (0.73–0.89)	
Age, years (SD)	74.2 ± 12.3	68.0 ± 16.0	-	<0.001
Comorbidities				
CVA, *n* (%)	259 (13.2)	863 (9.4)	1.47 (1.26–1.70)	<0.001
HTN, *n* (%)	1294 (65.8)	4601 (50.0)	1.92 (1.74–2.13)	<0.001
CAD, *n* (%)	230 (11.7)	660 (7.2)	1.71 (1.46–2.01)	<0.001
CHF, *n* (%)	53 (2.7)	130 (1.4)	1.93 (1.40–2.67)	<0.001
DM, *n* (%)	880 (44.7)	2335 (25.4)	2.38 (2.15–2.63)	<0.001
ESRD, *n* (%)	171 (8.7)	328 (3.6)	2.58 (2.13–3.12)	<0.001
GCS, median (IQR)	15 (15–15)	15 (15–15)	-	<0.001
3–8, *n* (%)	73 (3.7)	164 (1.8)	2.12 (1.61–2.81)	<0.001
9–12, *n* (%)	89 (4.5)	220 (2.4)	1.93 (1.51–2.49)	<0.001
13–15, *n* (%)	1806 (91.8)	8821 (95.8)	0.49 (0.40–0.59)	<0.001
ISS, median (IQR)	9 (9–9)	9 (4–9)	-	<0.001
1–15, *n* (%)	1624 (82.5)	8206 (89.1)	0.58 (0.50–0.66)	<0.001
16–24, *n* (%)	267 (13.6)	811 (8.8)	1.63 (1.40–1.88)	<0.001
≥25, *n* (%)	77 (3.9)	188 (2.0)	1.95 (1.49–2.56)	<0.001
Mortality, *n* (%)	96 (4.9)	186 (2.0)	2.49 (1.93–3.20)	<0.001
Hospital stay (days)	10.1 ± 11.4	7.1 ± 7.6	-	<0.001

CAD: coronary artery disease; CHF: congestive heart failure; CVA: cerebral vascular accident; CI: confidence interval; DM: diabetes mellitus; ESRD: end-stage renal disease; GCS: Glasgow Coma Scale; HTN: hypertension; IQR: interquartile range; ISS: injury severity score; OR: odds ratio; “-” indicates not able to calculate the data.

**Table 2:** The developed propensity scores-matched patient cohort of the research population in terms of the presence or absence of hyponatremia.

Propensity Score Matched-Cohort								
Variables	Hyponatremia							
	Yes *n* = 1903		No *n* = 1903		OR (95% CI)		*p*	SD
Male, *n* (%)	767	(40.3)	767	(40.3)	1.00	(0.88–1.14)	1.000	0.00%
Age, years (SD)	74.5	±12.0	74.5	±12.0	-		0.986	0.06%
CVA, *n* (%)	234	(12.3)	234	(12.3)	1.00	(0.82–1.21)	1.000	0.00%
HTN, *n* (%)	1251	(65.7)	1251	(65.7)	1.00	(0.88–1.14)	1.000	0.00%
CAD, *n* (%)	208	(10.9)	208	(10.9)	1.00	(0.82–1.23)	1.000	0.00%
CHF, *n* (%)	34	(1.8)	34	(1.8)	1.00	(0.62–1.62)	1.000	0.00%
DM, *n* (%)	836	(43.9)	836	(43.9)	1.00	(0.88–1.14)	1.000	0.00%
ESRD, *n* (%)	124	(6.5)	124	(6.5)	1.00	(0.77–1.29)	1.000	0.00%

CAD: coronary artery disease; CHF: congestive heart failure; CVA: cerebral vascular accident; CI: confidence interval; DM: diabetes mellitus; ESRD: end-stage renal disease; HTN: hypertension; OR: odds ratio; SD: standardized difference; “-” indicates not able to calculate the data.

**Table 3:** The incidences of fracture in body location in propensity scores-matched patient cohort in terms of the presence or absence of hyponatremia.

Variables	Hyponatremia		OR (95% CI)	*p*
	Yes *n* = 1903	No *n* = 1903		
Head trauma				
Cranial fracture, *n* (%)	50 (2.6)	54 (2.8)	0.92 (0.63–1.37)	0.691
Cervical vertebral fracture, *n* (%)	11 (0.6)	10 (0.5)	1.10 (0.47–2.60)	0.827
Maxillofacial trauma				
Orbital fracture, *n* (%)	9 (0.5)	9 (0.5)	1.00 (0.40–2.53)	1.000
Nasal fracture, *n* (%)	4 (0.2)	1 (0.1)	4.01 (0.45–35.88)	0.179
Maxillary fracture, *n* (%)	14 (0.7)	18 (0.9)	0.78 (0.39–1.57)	0.478
Mandibular fracture, *n* (%)	7 (0.4)	4 (0.2)	1.75 (0.51–6.00)	0.365
Thoracic trauma				
Rib fracture, *n* (%)	59 (3.1)	44 (2.3)	1.35 (0.91–2.01)	0.134
Thoracic vertebral fracture, *n* (%)	66 (3.5)	41 (2.2)	1.63 (1.10–2.42)	0.014
Abdominal trauma				
Lumbar vertebral fracture, *n* (%)	34 (1.8)	24 (1.3)	1.42 (0.84–2.41)	0.186
Extremity trauma				
Scapular fracture, *n* (%)	10 (0.5)	4 (0.2)	2.51 (0.79–8.01)	0.108
Clavicle fracture, *n* (%)	26 (1.4)	23 (0.2)	1.13 (0.64–1.99)	0.666
Humeral fracture, *n* (%)	109 (5.7)	111 (5.8)	0.98 (0.75–1.29)	0.890
Radial fracture, *n* (%)	144 (7.6)	219 (11.5)	0.63 (0.51–0.79)	<0.001
Ulnar fracture, *n* (%)	68 (3.6)	90 (4.7)	0.75 (0.54–1.03)	0.074
Metacarpal fracture, *n* (%)	9 (0.5)	13 (0.7)	0.69 (0.30–1.62)	0.392
Pelvic fracture, *n* (%)	25 (1.3)	11 (0.6)	2.29 (1.12–4.67)	0.019
Femoral fracture, *n* (%)	932 (49.0)	816 (42.9)	1.28 (1.13–1.45)	<0.001
Patella fracture, *n* (%)	44 (2.3)	69 (3.6)	0.63 (0.43–0.92)	0.017
Tibia fracture, *n* (%)	34 (1.8)	37 (1.9)	0.92 (0.57–1.47)	0.719
Fibular fracture, *n* (%)	22 (1.2)	17 (0.9)	1.30 (0.69–2.45)	0.421
Calcaneal fracture, *n* (%)	48 (2.5)	54 (2.8)	0.89 (0.60–1.31)	0.547
Metatarsal fracture, *n* (%)	15 (0.8)	11 (0.6)	1.37 (0.63–2.98)	0.431

CI: confidence interval; OR: odds ratio.

**Table 4:** Demographic and injury characteristics of adult trauma patients admitted to the hospital—a comparison with and without hypokalemia.

	Hypokalemia			
Variables	Yes *n* = 1986	No *n* = 9187	OR (95% CI)	*p*
Sex				0.334
Male, *n* (%)	702 (35.3)	3353 (36.5)	0.95 (0.86–1.05)	
Female, *n* (%)	1284 (64.7)	5834 (63.5)	1.05 (0.95–1.16)	
Age, years (SD)	69.1 ± 15.4	69.1 ± 15.6	-	0.981
Comorbidities				
CVA, *n* (%)	230 (11.6)	892 (9.7)	1.22 (1.04–1.42)	0.012
HTN, *n* (%)	1153 (58.1)	4742 (51.6)	1.30 (1.18–1.43)	<0.001
CAD, *n* (%)	148 (7.5)	742 (8.1)	0.92 (0.76–1.10)	0.351
CHF, *n* (%)	31 (1.6)	152 (1.7)	0.94 (0.64–1.39)	0.766
DM, *n* (%)	470 (23.7)	2745 (29.9)	0.73 (0.65–0.81)	<0.001
ESRD, *n* (%)	66 (3.3)	433 (4.7)	0.70 (0.53–0.91)	0.007
GCS, median (IQR)	15 (15–15)	15 (15–15)	-	<0.001
3–8, *n* (%)	101 (5.1)	136 (1.5)	3.57 (2.74–4.64)	<0.001
9–12, *n* (%)	72 (3.6)	237 (2.6)	1.42 (1.09–1.86)	0.010
13–15, *n* (%)	1813 (91.3)	8814 (95.9)	0.44 (0.37–0.54)	<0.001
ISS, median (IQR)	9 (4–9)	9 (4–9)	-	<0.001
1–15, *n* (%)	1622 (81.7)	8208 (89.3)	0.53 (0.47–0.61)	<0.001
16–24, *n* (%)	274 (13.8)	804 (8.8)	1.67 (1.44–1.93)	<0.001
≥25, *n* (%)	90 (4.5)	175 (1.9)	2.44 (1.89–3.17)	<0.001
Mortality, *n* (%)	84 (4.2)	198 (2.2)	2.01 (1.55–2.60)	<0.001
Hospital stay (days)	8.7 ± 10.3	7.4 ± 8.0	-	<0.001

CAD: coronary artery disease; CHF: congestive heart failure; CVA: cerebral vascular accident; CI: confidence interval; DM: diabetes mellitus; ESRD: end-stage renal disease; GCS: Glasgow Coma Scale; HTN: hypertension; IQR: interquartile range; ISS: injury severity score; OR: odds ratio; “-” indicates not able to calculate the data.

**Table 5:** The developed propensity scores-matched patient cohort of the research population in terms of the presence or absence of hypokalemia.

Propensity Score Matched-Cohort								
	Hypokalemia							
	Yes *n* = 1977		No *n* = 1977		OR (95% CI)		*p*	SD
Male, *n* (%)	699	(35.4)	699	(35.4)	1.00	(0.88–1.14)	1.000	0.00%
Age, years (SD)	69.1	±15.4	69.2	±15.3	-		0.894	−0.43%
CVA, *n* (%)	224	(11.3)	224	(11.3)	1.00	(0.82–1.22)	1.000	0.00%
HTN, *n* (%)	1149	(58.1)	1149	(58.1)	1.00	(0.88–1.14)	1.000	0.00%
CAD, *n* (%)	144	(7.3)	144	(7.3)	1.00	(0.79–1.27)	1.000	0.00%
CHF, *n* (%)	28	(1.4)	28	(1.4)	1.00	(0.59–1.70)	1.000	0.00%
DM, *n* (%)	467	(23.6)	467	(23.6)	1.00	(0.86–1.16)	1.000	0.00%
ESRD, *n* (%)	62	(3.1)	62	(3.1)	1.00	(0.70–1.43)	1.000	0.00%

CAD: coronary artery disease; CHF: congestive heart failure; CVA: cerebral vascular accident; CI: confidence interval; DM: diabetes mellitus; ESRD: end-stage renal disease; HTN: hypertension; OR: odds ratio; SD: standardized difference; “-” indicates not able to calculate the data.

**Table 6:** The incidences of fracture in body location in propensity scores-matched patient cohort in terms of the presence or absence of hypokalemia.

Variables	Hypokalemia		OR (95% CI)	*p*
	Yes *n* = 1977	No *n* = 1977		
Head trauma				
Cranial fracture, *n* (%)	81 (4.1)	77 (3.9)	1.14 (0.92–1.23)	0.538
Cervical vertebral fracture, *n* (%)	15 (0.8)	11 (0.6)	1.37 (0.63–2.98)	0.431
Maxillofacial trauma				
Orbital fracture, *n* (%)	16 (0.8)	10 (0.5)	1.61 (0.73–3.55)	0.238
Nasal fracture, *n* (%)	8 (0.4)	5 (0.3)	1.60 (0.52–4.91)	0.405
Maxillary fracture, *n* (%)	24 (1.2)	26 (1.3)	0.92 (0.53–1.61)	0.776
Mandibular fracture, *n* (%)	13 (0.7)	14 (0.7)	0.93 (0.44–1.98)	0.847
Thoracic trauma				
Rib fracture, *n* (%)	50 (2.5)	57 (2.9)	0.87 (0.60–1.28)	0.493
Thoracic vertebral fracture, *n* (%)	25 (1.3)	18 (0.9)	1.39 (0.76–2.56)	0.283
Abdominal trauma				
Lumbar vertebral fracture, *n* (%)	30 (1.5)	23 (1.2)	1.31 (0.76–2.26)	0.333
Extremity trauma				
Scapular fracture, *n* (%)	6 (0.3)	2 (0.1)	3.01 (0.61–14.91)	0.157
Clavicle fracture, *n* (%)	30 (1.5)	35 (1.8)	0.86 (0.52–1.40)	0.532
Humeral fracture, *n* (%)	113 (5.7)	99 (5.0)	1.15 (0.87–1.52)	0.323
Radial fracture, *n* (%)	213 (10.8)	305 (15.4)	0.66 (0.55–0.80)	<0.001
Ulnar fracture, *n* (%)	91 (4.6)	104 (5.3)	0.87 (0.65–1.16)	0.340
Metacarpal fracture, *n* (%)	8 (0.4)	11 (0.6)	0.73 (0.29–1.81)	0.490
Pelvic fracture, *n* (%)	15 (0.8)	11 (0.6)	1.37 (0.63–2.98)	0.431
Femoral fracture, *n* (%)	779 (39.4)	744 (37.6)	1.08 (0.95–1.23)	0.253
Patella fracture, *n* (%)	73 (3.7)	97 (4.9)	0.74 (0.55–1.01)	0.060
Tibia fracture, *n* (%)	49 (2.5)	52 (2.6)	0.94 (0.63–1.40)	0.762
Fibular fracture, *n* (%)	24 (1.2)	33 (1.7)	0.72 (0.43–1.23)	0.230
Calcaneal fracture, *n* (%)	68 (3.4)	90 (4.6)	0.75 (0.54–1.03)	0.074
Metatarsal fracture, *n* (%)	21 (1.1)	25 (1.3)	0.84 (0.47–1.50)	0.553

CI: confidence interval; OR: odds ratio.

**Table 7:** Demographic and injury characteristics of adult trauma patients admitted to the hospital—a comparison with and without both hyponatremia and hypokalemia.

Variables	Hyponatremia and Hypokalemia *n* = 385	Normal *n* = 7601	OR (95% CI)	*p*
Sex				0.001
Male, *n* (%)	169 (43.9)	2724 (35.8)	1.40 (1.14–1.72)	
Female, *n* (%)	216 (56.1)	4880 (64.2)	0.71 (0.58–0.88)	
Age, years (SD)	72.0 ± 13.7	67.9 ± 16.1	-	<0.001
Comorbidities				
CVA, *n* (%)	39 (10.1)	672 (8.8)	1.16 (0.83–1.63)	0.385
HTN, *n* (%)	250 (64.9)	3698 (48.6)	1.96 (1.58–2.42)	<0.001
CAD, *n* (%)	43 (11.2)	555 (7.3)	1.60 (1.15–2.22)	0.005
CHF, *n* (%)	7 (1.8)	106 (1.4)	1.31 (0.61–2.83)	0.492
DM, *n* (%)	127 (33.0)	1992 (26.2)	1.40 (1.12–1.73)	0.003
ESRD, *n* (%)	22 (5.7)	284 (3.7)	1.56 (1.00–2.44)	0.048
GCS, median (IQR)	15 (15–15)	15 (15–15)	-	<0.001
3–8, *n* (%)	31 (8.1)	94 (1.2)	7.00 (4.60–10.65)	<0.001
9–12, *n* (%)	20 (5.2)	168 (2.2)	2.43 (1.51–3.90)	<0.001
13–15, *n* (%)	334 (86.8)	7342 (96.6)	0.23 (0.17–0.32)	<0.001
ISS, median (IQR)	9 (9–16)	9 (4–9)	-	<0.001
1–15, *n* (%)	280 (72.7)	6864 (90.3)	0.29 (0.23–0.36)	<0.001
16–24, *n* (%)	80 (20.8)	617 (8.1)	2.97 (2.29–3.85)	<0.001
≥25, *n* (%)	25 (6.5)	123 (1.6)	4.22 (2.71–6.58)	<0.001
Mortality, *n* (%)	28 (7.3)	130 (1.7)	4.51 (2.96–6.88)	<0.001
Hospital stay (days)	11.6 ± 14.7	6.9 ± 7.3	-	<0.001

CAD: coronary artery disease; CHF: congestive heart failure; CVA: cerebral vascular accident; CI: confidence interval; DM: diabetes mellitus; ESRD: end-stage renal disease; GCS: Glasgow Coma Scale; HTN: hypertension; IQR: interquartile range; ISS: injury severity score; OR: odds ratio; “-” indicates not able to calculate the data.

**Table 8:** The developed propensity scores-matched patient cohort of the research population in terms of the presence or absence of both hyponatremia and hypokalemia.

Propensity Score Matched-Cohort								
	Hyponatremia and Hypokalemia *n* = 380		Normal *n* = 380		OR (95% CI)		*p*	SD
Male, *n* (%)	166	(43.7)	166	(43.7)	1.00	(0.75–1.33)	1.000	0.00%
Age, years (SD)	72.1	±13.6	72.2	±13.6	-		0.932	−0.62%
CVA, *n* (%)	37	(9.7)	37	(9.7)	1.00	(0.62–1.62)	1.000	0.00%
HTN, *n* (%)	248	(65.3)	248	(65.3)	1.00	(0.74–1.35)	1.000	0.00%
CAD, *n* (%)	39	(10.3)	39	(10.3)	1.00	(0.63–1.60)	1.000	0.00%
CHF, *n* (%)	6	(1.6)	6	(1.6)	1.00	(0.32–3.13)	1.000	0.00%
DM, *n* (%)	123	(32.4)	123	(32.4)	1.00	(0.74–1.36)	1.000	0.00%
ESRD, *n* (%)	18	(4.7)	18	(4.7)	1.00	(0.51–1.95)	1.000	0.00%

CAD: coronary artery disease; CHF: congestive heart failure; CVA: cerebral vascular accident; CI: confidence interval; DM: diabetes mellitus; ESRD: end-stage renal disease; HTN: hypertension; OR: odds ratio; SD: standardized difference; “-” indicates not able to calculate the data.

**Table 9:** The incidences of fracture in body location in propensity scores-matched patient cohort in terms of the presence or absence of both hyponatremia and hypokalemia.

Variables	Hyponatremia and Hypokalemia *n* = 380	Normal *n* = 380	OR (95% CI)	*p*
Head trauma				
Cranial fracture, *n* (%)	18 (4.7)	12 (3.2)	1.53 (0.72–3.21)	0.264
Cervical vertebral fracture, *n* (%)	3 (0.8)	5 (1.3)	0.60 (0.14–2.52)	0.477
Maxillofacial trauma				
Orbital fracture, *n* (%)	1 (0.3)	3 (0.8)	0.33 (0.03–3.20)	0.316
Nasal fracture, *n* (%)	0 (0.0)	3 (0.8)	-	0.083
Maxillary fracture, *n* (%)	1 (0.3)	1 (0.3)	1.00 (0.06–16.05)	1.000
Mandibular fracture, *n* (%)	3 (0.8)	1 (0.3)	3.02 (0.31–29.12)	0.316
Thoracic trauma				
Rib fracture, *n* (%)	16 (4.2)	12 (3.2)	1.35 (0.63–2.89)	0.441
Thoracic vertebral fracture, *n* (%)	7 (1.8)	4 (1.1)	1.76 (0.51–6.08)	0.362
Abdominal trauma				
Lumbar vertebral fracture, *n* (%)	3 (0.8)	2 (0.5)	1.50 (0.25–9.05)	0.654
Extremity trauma				
Scapular fracture, *n* (%)	1 (0.3)	1 (0.3)	1.00 (0.06–16.05)	1.000
Clavicle fracture, *n* (%)	9 (2.4)	5 (1.3)	1.82 (0.60–5.48)	0.281
Humeral fracture, *n* (%)	23 (6.1)	22 (5.8)	1.05 (0.57–1.92)	0.878
Radial fracture, *n* (%)	29 (7.6)	54 (14.2)	0.50 (0.31–0.80)	0.004
Ulnar fracture, *n* (%)	12 (3.2)	21 (5.5)	0.56 (0.27–1.15)	0.109
Metacarpal fracture, *n* (%)	1 (0.3)	3 (0.8)	0.33 (0.03–3.20)	0.316
Pelvic fracture, *n* (%)	6 (1.6)	1 (0.3)	6.08 (0.73–50.75)	0.058
Femoral fracture, *n* (%)	163 (42.9)	162 (42.6)	1.01 (0.76–1.35)	0.942
Patella fracture, *n* (%)	4 (1.1)	18 (4.7)	0.21 (0.07–0.64)	0.002
Tibia fracture, *n* (%)	7 (1.8)	10 (2.6)	0.69 (0.26–1.84)	0.462
Fibular fracture, *n* (%)	4 (1.1)	8 (2.1)	0.50 (0.15–1.66)	0.244
Calcaneal fracture, *n* (%)	4 (1.1)	11 (2.9)	0.36 (0.11–1.13)	0.068
Metatarsal fracture, *n* (%)	2 (0.5)	2 (0.5)	1.00 (0.14–7.14)	1.000

CI: confidence interval; OR: odds ratio; “-” indicates not able to calculate the data.

**Table 10:** A summary of the risk of bone fractures in adult trauma patients who are hospitalized and have hyponatremia and/or hypokalemia.

Hyponatremia	Hyponatremia and Hypokalemia	Hypokalemia
Radial fracture ↓	Radial fracture ↓	Radial fracture ↓
Patella fracture ↓	Patella fracture ↓	Patella fracture ↓
Thoracic vertebral fracture ↑		
Pelvic fracture ↑		
Femoral fracture ↑		

↓: decreased incidence of occurrence; ↑: increase incidence of occurrence.

## Data Availability

De-identification data available on request.
